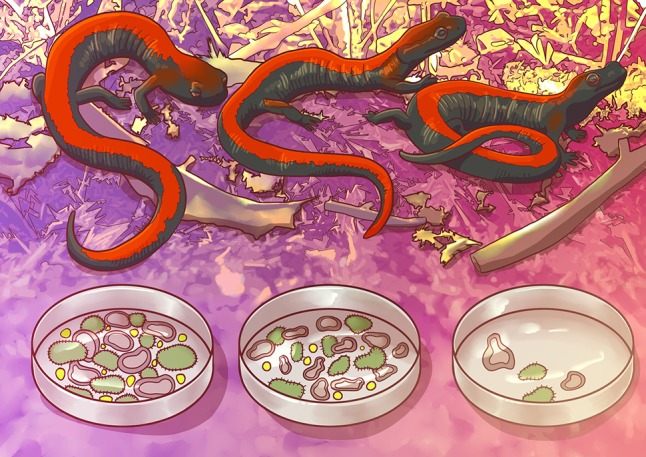# Too hot for a healthy gut in salamanders

**DOI:** 10.1093/conphys/coz007

**Published:** 2019-02-27

**Authors:** Rachael M Heuer

**Affiliations:** University of Miami, Rosenstiel School of Marine and Atmospheric Science, 4600 Rickenbacker Causeway, Miami, FL, USA

Have you ever taken a probiotic? Society spends millions of dollars every year on supplements containing ‘healthy bacteria’ in an attempt to maintain a healthy digestive system. Not surprisingly, a healthy gut microbiome is important for many other species as well.

A new study by Samantha Fontaine and her team examines how environmental factors like temperature can influence the gut microbiome and digestive performance of eastern red-backed salamanders. Unlike humans, who generally maintain a constant internal body temperature, salamanders are ectotherms, so their internal temperature is similar to that of their surrounding environment. This means that the temperature of their internal organs, like their gut, will be strongly influenced by environmental temperatures. Conditions that are too cold or too hot can negatively impact physiological processes.

To understand how temperature affects salamander gut bacteria, the team acclimated salamanders to three different temperatures: two spanning the lowest and highest ends of their nocturnal summer range (10 and 20°C) and one in the middle (15°C). Then, the team measured digestive performance in salamanders. They did this by feeding them a known number of fruit flies, and then they measured how this food was converted to energy by examining feces and shed skin. As the team expected, the salamanders that were acclimated to the intermediate temperature had the best digestive performance.

Next, Fontaine’s team wanted to examine what kind of bacteria were living in the gut. So, they collected feces from salamanders at each temperature and performed DNA analysis to identify the bacteria present in each sample. Using this technique, the team found that salamanders from the highest temperature had fewer bacteria species (i.e., lower biodiversity) when compared to salamanders from the other temperature groups.

With DNA results in hand, Fontaine’s team did a little more digging and found some interesting trends when looking at the types of bacteria they found. They determined that several of the bacteria that are known to break down carbohydrates that are typical in insect diets were high in salamanders maintained at the intermediate temperature. This was also the same temperature where digestive performance was enhanced. The guts from salamanders maintained at the high temperatures, unfortunately, showed a reduction in the bacteria known to fend off chytridiomycosis, a fungus that is deadly for many amphibians. The team concluded that, for salamanders living at high temperatures, lower gut biodiversity, in addition to reductions in healthy gut bacteria, might lead to increased vulnerability to pathogens and disease.

So why does this temperature effect matter? Fontaine and her team point out that ectotherms are considered to be one of the animal groups most sensitive to climate change. Changes to gut microbial diversity may be yet another factor that could reduce the health and physiological performance of salamanders in a warmer world. Fortunately, Fontaine and her research team unearthed this important factor so we can study it further. If we know more about the link between temperature and the gut microbiome, we can better assess the health of this important group of organisms.

Illustration by Erin Walsh; Email: ewalsh.sci@gmail.com

**Figure:**